# LAD-GCN: Automatic diagnostic framework for quantitative estimation of growth patterns during clinical evaluation of lung adenocarcinoma

**DOI:** 10.3389/fphys.2022.946099

**Published:** 2022-08-10

**Authors:** Wei Xiao, Yanyun Jiang, Zhigang Yao, Xiaoming Zhou, Xiaodan Sui, Yuanjie Zheng

**Affiliations:** ^1^ Shandong Provincial Hospital, Shandong University, Jinan, China; ^2^ School of Information Science and Engineering, Shandong Normal University, Jinan, China; ^3^ Department of Pathology, Shandong Provincial Hospital Affiliated to Shandong First Medical University Department of Pathology, Shandong Provincial Hospital, Cheeloo College of Medicine, Shandong University, Jinan, China

**Keywords:** lung adenocarcinoma, histopathological, deep learning, polar representation-based model, graph convolutional networks, feature fusion

## Abstract

Quantitative estimation of growth patterns is important for diagnosis of lung adenocarcinoma and prediction of prognosis. However, the growth patterns of lung adenocarcinoma tissue are very dependent on the spatial organization of cells. Deep learning for lung tumor histopathological image analysis often uses convolutional neural networks to automatically extract features, ignoring this spatial relationship. In this paper, a novel fully automated framework is proposed for growth pattern evaluation in lung adenocarcinoma. Specifically, the proposed method uses graph convolutional networks to extract cell structural features; that is, cells are extracted and graph structures are constructed based on histopathological image data without graph structure. A deep neural network is then used to extract the global semantic features of histopathological images to complement the cell structural features obtained in the previous step. Finally, the structural features and semantic features are fused to achieve growth pattern prediction. Experimental studies on several datasets validate our design, demonstrating that methods based on the spatial organization of cells are appropriate for the analysis of growth patterns.

## 1 Introduction

Lung cancer is a malignant tumor originating from the bronchial mucosa or glands of the lungs that poses a great threat to human health and life. In recent years, many countries have reported significant increases in rates of lung cancer; in men, lung cancer has the highest morbidity and mortality among all malignant tumors (Ferlay et al., 2020). The 5-year survival rate of patients with lung cancer is relatively low at 19%, mainly owing to the high risk of distant metastasis (Hirsch et al., 2017; Mayekar and Bivona, 2017). Adenocarcinoma is the most common histopathological type of lung cancer and accounts for up to 40% of lung cancer cases (Cheng et al., 2016).

**FIGURE 1:**
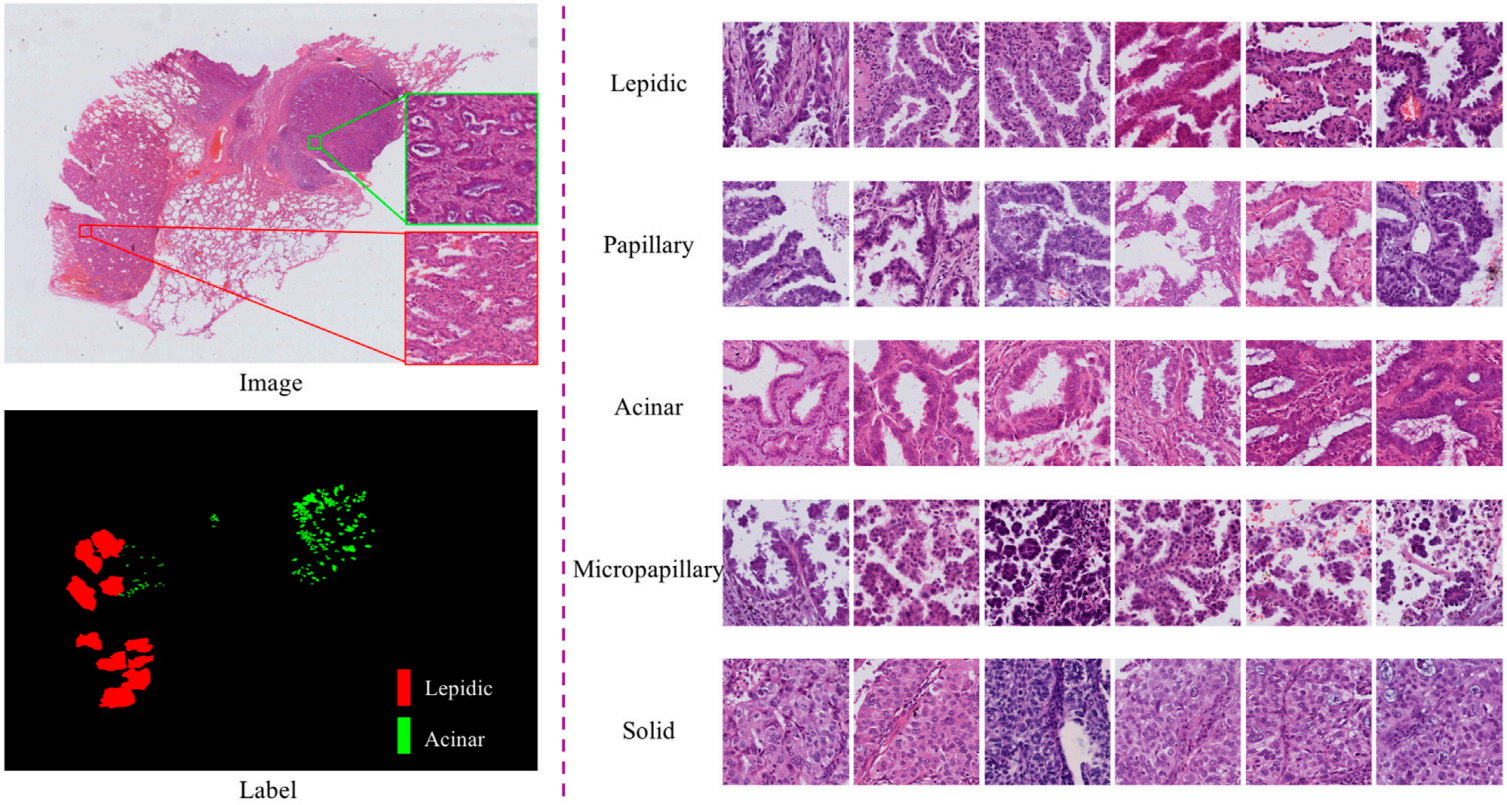
Pictures of typical growth patterns. Left: An example of an HandE-stained digital pathology image with manual segmentation of growth patterns, where red is the lepidic growth pattern and green is the acinar growth pattern. Right: Five common growth patterns (lepidic, papillary, acinar, micropapillary, and solid).

According to the 2011 IASLC/ATS/ERS lung adenocarcinoma classification, lung adenocarcinoma has five predominant growth patterns: lepidic, papillary, acinar, micropapillary, and solid (Travis et al., 2011). Accurate determination of growth patterns and proportions from whole-slide images (WSIs) has crucial clinical implications for diagnosis, subsequent treatment, and prognosis. The World Health Organization recommends that invasive adenocarcinoma should be semi-quantitatively estimated in 5% increments with respect to the various growth patterns on histopathological slides (Travis et al., 2015). Typically, this work involves visual inspection by experienced pathologists through a microscope, which is a very time-consuming and labor-intensive process (Gurcan et al., 2009). In particular, semi-quantitative estimates of growth patterns are required; however, traditional methods only allow estimation and not quantification. WSI technology and computer-aided diagnosis provide an effective strategy for lung adenocarcinoma diagnosis, which can be used as an auxiliary basis for manual evaluation and to alleviate the shortage of pathologists. In this work, we focus on the identification and quantification of lung adenocarcinoma tissue growth patterns from WSIs. This is expected to help pathologists to make rapid diagnoses in practical clinical applications and provide a basis for subsequent treatment. However, identifying growth patterns is challenging because of the high intraclass variation and low interclass distinction among patterns. As shown in, Figure 1, the spatial structure of lung cancer cells has complicated characteristic manifestations; for instance, cells in the lepidic growth pattern grow along alveolar walls in a lepidic fashion, and the acinar growth pattern has well-defined individual tumor glands with well-formed glandular lumina.

Recently, owing to the enormous potential of deep learning, many convolutional neural network (CNN)-based methods have emerged that can automatically extract more beneficial features for classification compared with hand-crafted features (Szegedy et al., 2016; Coudray et al., 2018; Šarić et al., 2019; Noorbakhsh et al., 2020; Yu et al., 2020; Fan et al., 2021). For example, Coudray et al. (2018) used an Inception v3 architecture (Szegedy et al., 2016) to learn a parametric function to automatically classify lung tumor subtypes (adenocarcinoma and squamous cell carcinoma) and predict mutations using a dataset from The Cancer Genome Atlas. Yu et al. (2020) built a CNN model to identify tumor regions from whole-slide histopathology images, achieving an area under the curve value (AUC) > 0.935, and used the proposed model to predict pathologists’ diagnoses. Kalra et al. (2020) presented a memory-based exchangeable model that could learn interdependencies among instances through a self-attention mechanism, achieving a competitive accuracy of 84.84% for the classification of lung adenocarcinoma and squamous cell carcinoma. Although CNN can automatically encode rich semantic features contained in captured images, the analysis of histopathological images often focuses on local features, which leads to biased learning. The main reason for this is that the interclass differences among tissue growth patterns are small, that is, cancer cells with different tissue growth patterns show no obvious visual differences. Furthermore, intraclass differences in tissue growth patterns are evident, and models need to cope with altered tumor tissue and cell diversity. Therefore, as well as understanding the semantic features of an image, an algorithm designed for classification of lung tumor histopathological images needs to be able to analyze the spatial structure between cancer cells. Graph convolutional networks (GCN) (Kipf and Welling, 2016), variants of CNN that transform data into spatially structured features, have recently become a popular choice for processing structured data in the field of computer vision. Some studies have proposed the use of GCN to analyze histopathological images. For example, Li et al. (2018) proposed a GCN for WSIs, which obtains graph nodes by sampling representative patches and extracting features for survival prediction. Adnan et al. (2020) introduced attention through graph pooling to infer relations among sampled patches and applied multiple instance learning to classify lung cancer subtypes. Zhou et al. (2019) developed CGC-Net, which converts a large histology image into a graph, where each graph node is a nucleus and the connecting edges of the nodes represent the similarity between nuclei. Wang et al. (2020) also segmented the cell nucleus and extracted topological composition graphs for tumor microenvironment analysis in renal cell carcinoma and patient outcome prediction; however, they did not use GCN.

Inspired by the above methods, in this work we designed a novel deep learning framework, called LAD-GCN (lung adenocarcinoma diagnosis GCN), which aggregates the advantages of GCN and CNN for analyzing histopathology. Specifically, to capture complex tumor microenvironment information and semantic information of entire image patches, we designed a model with two independent feature extraction branches as follows. 1) The GCN module, including a polar representation-based instance segmentation model (Xiao et al., 2021), is used to extract all the cell nuclei contained in the histopathological patch and extract a nuclear feature composition map, which is used as an input to the GCN network to extract cell structural features. 2) The CNN module directly extracts semantic information from the whole patch to supplement the information loss of the GCN module. Then, the cell structural features extracted by the GCN branch and the image patch semantic features extracted by the CNN branch are fused. Compared with the CNN-only models that are widely used in image classification tasks, LAD-GCN could provide complementary semantic and cell structural information during feature extraction. Finally, we quantitatively evaluated the proposed method on a private dataset of lung adenocarcinoma postoperative formalin-fixed, paraffin-embedded (FFPE) tissue slides. The results demonstrate that our method is able to capture features that are beneficial for growth pattern typing. Our major contributions can be summarized as follows.

1. In response to the problem of the small interclass differences in tissue growth patterns that mean there are no obvious visual differences among cancer cells with different growth patterns, we developed a novel GCN-based framework for analysis of the histopathological growth patterns of lung adenocarcinoma. The proposed method adopts a polar representation-based instance segmentation model to segment the nucleus and uses GCN to extract cell spatial structural features.
2. To overcome the limitations of a single feature extraction module, we designed a dual-network joint analysis method: the GCN branch extracts the spatial structural features of cells, while the CNN branch complements these with the extraction of semantic features of patches.
3. We validated the proposed method on a private lung adenocarcinoma WSI dataset, demonstrating the effectiveness of the architecture.

## 2 Materials and methods

### 2.1 Materials

Our histopathological image dataset contained data obtained from 243 lung adenocarcinoma patients at Shandong Provincial Hospital; for each patient, there was one FFPE image of the tumor area, stained with hematoxylin and eosin (HandE) and scanned at 20× and 40× magnification with a pixel scale of 0.23 μm × 0.23 μm. All samples represented postoperative pathology, including tumor tissue slides, normal tissue slides, and slides containing the border between normal and tumor tissue. In this dataset, all data were positive samples, that is, slides containing tumor tissue. The tumor/non-tumor area and five histological patterns (lepidic, acinar, papillary, micropapillary, and solid) were manually delineated by an experienced oncology pathologist.

**FIGURE 2:**
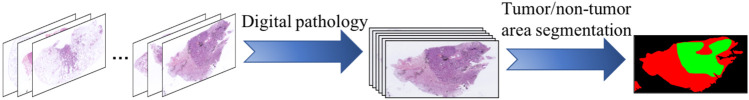
Schematic representation of data processing; we used U-Net as the backbone to segment tumor regions.

To make the algorithm more effective, we built a segmentation model based on U-Net (Ronneberger et al., 2015) to achieve tumor region extraction; the process is shown in Figure 2. Specifically, for the images in the dataset, both tumor and non-tumor regions were manually annotated by pathologists. We derived 2× magnification WSIs, which were full-coverage images, and trained the U-Net backbone in a traditional fully supervised manner with a cross-entropy loss function (Xie and Tu, 2015) to predict tumor regions in the WSIs. It is worth noting that the size of the original pathological images was non-uniform, and the model was able to achieve region prediction in images of any size.

### 2.2 Overview of the LAD-GCN architecture

**FIGURE 3:**
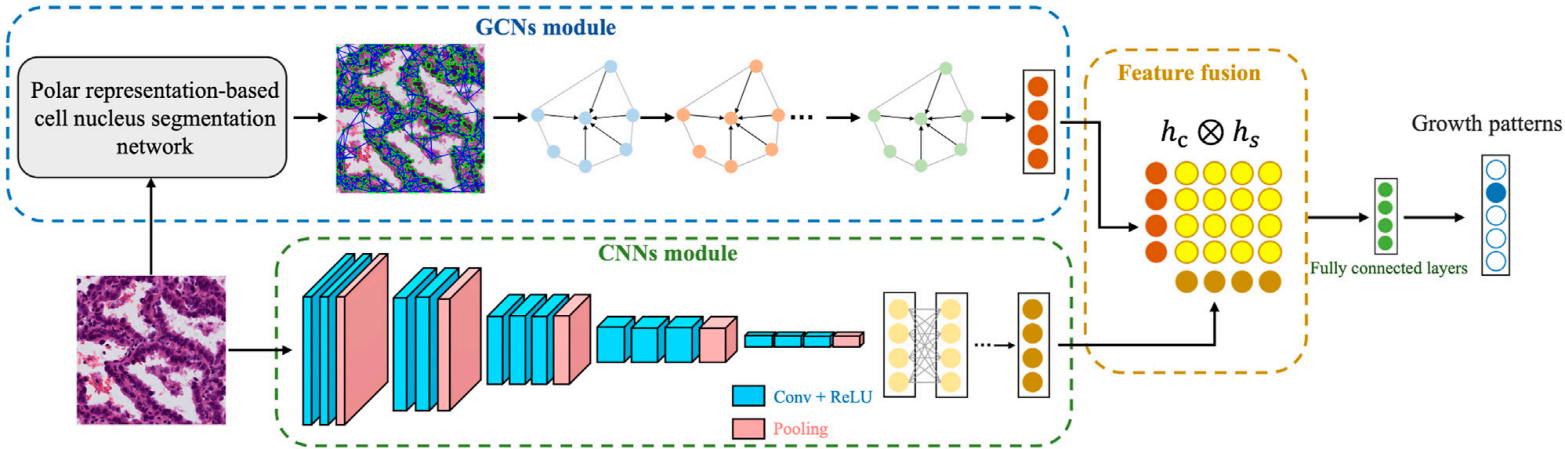
Overview of the proposed diagnostic methods for lung adenocarcinoma.

Figure 3 provides an overview of our automatic diagnosis framework. As shown in the figure, instead of directly extracting features using a CNN, we developed both CNN and GCN feature extractors simultaneously. The inputs are patches from digitized postoperative FFPE tissue slides, and the output is the predicted growth pattern type. The whole process consists of three parts. 1) GCN module: a polar representation-based instance segmentation model is used to segment all the cell nuclei contained in the histopathological patch; the nuclear features are extracted to form a composite map that can be used as the input to the GCN; and then GCN are used to extract cell spatial structure features. 2) CNN module: semantic feature extraction is performed using a CNN, VGG16 (Simonyan and Zisserman, 2015). 3) Feature fusion: cell spatial structural features and semantic features are fused for tumor growth pattern prediction.

### 2.3 Spatial feature encoding with GCN

In histopathology images, each cell has its own characteristic information, and there is structural information between cells. To extract this information, we segment out the nuclei and construct a graph of the tumor microenvironment for graph convolution operations. Specifically, we first extract all the nuclei contained in the patch and calculate the centroids of the nuclei to define the graph node set *V*; then extract the nuclei features, use K-nearest neighbors (KNN) (Muja and Lowe, 2009) to find the connections between adjacent cells to define the edge set *A* (Chen et al., 2020); and, finally, use GCN to learn the graph depth spatial structural features.

#### 2.3.1 Nuclei segmentation module

**FIGURE 4:**
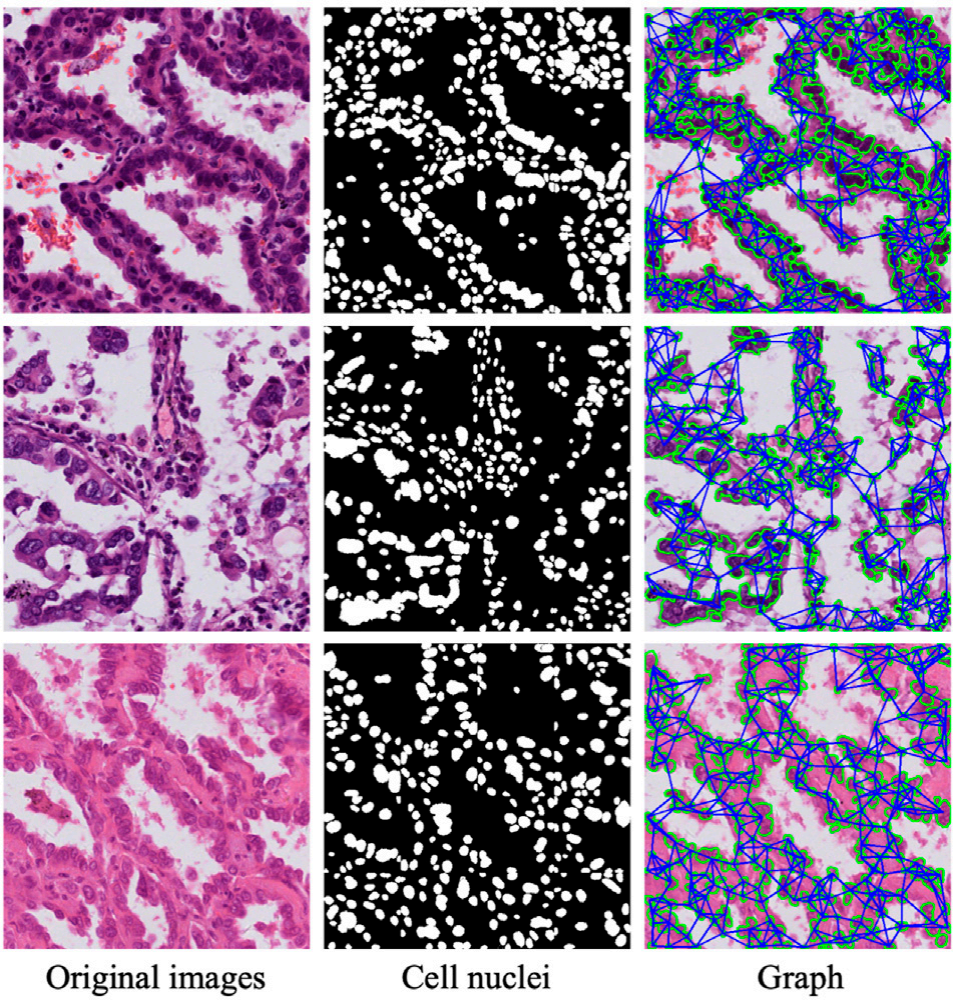
Three sample patches of cell nuclei structures. First column: typical patches from lung tumor histopathological images. Second column: nuclei segmentation mask from nuclei segmentation module. Third column: graph nodes and edges.

The major purpose of the nuclei segmentation module is to extract the various nuclei contained in the input image patch, which includes normal cells, tumor cells, and stromal cells. The cell nuclei produced by the nuclei segmentation module are then constructed as a graph and fed into a GCN module for spatial feature extraction. To achieve this aim, we use a polar representation-based instance segmentation model (Xiao et al., 2021) from our previous work to learn the segmentation of nuclei; this model leverages fully convolutional one-stage object detection and consists of a backbone network, feature pyramid network, and task-specific heads. Specifically, when we input an original image via the proposed network, the position of the cell center point and the distance of n (*n* = 36) root rays can be obtained; then, the coordinates of these points on the contour are calculated according to the angle and length, connecting these points starting from 0°; and, finally, the regions within the connected regions are taken as the results of instance segmentation. The nuclei segmentation module models a contour based on the polar coordinate system and transforms the instance segmentation problem into an instance center classification problem and a dense distance regression problem (Xie et al., 2020); thus, the network only needs to return to the length of the fixed angle, which reduces the difficulty of the problem. Through the prediction of the segmentation module, we obtain the mask of the nuclei, and the second column in Figure 4 shows the result of the nuclei segmentation.

#### 2.3.2 Cell feature extraction and construct graph

A feature matrix for graph convolution is generated based on the nuclear segmentation map generated in the previous step. It is used for two main processes: cell feature extraction and graph construction. In the first of these processes, the PyRadiomics package (Van Griethuysen et al., 2017) in Python is used to generate features corresponding to each cell, including eight shape features and four textural features. The shape features include major axis length, minor axis length, angular orientation, eccentricity, roundness, area, and solidity. The textural features, obtained from gray-level co-occurrence matrices, are dissimilarity, homogeneity, angular second moment, and energy. In addition, we use contrastive predictive coding (Henaff, 2020) to encode features of 64 × 64 image patch regions centered on the centroids of cell nuclei.

In the second process, we connect the nuclei into a graph, using the centroid of each nucleus as a graph node, and use the KNN algorithm to build the edge set *A* of the graph. Specifically, in principle, each nucleus should have contact with the other nuclei, and the nearest neighbor cells are considered to have obvious intercellular interactions. The adjacency matrix is defined as:

$$a_{ij}=\left\{\begin{array}{c} 1,\text{if }j\in \text{KNN}\left(i\right)\text{ and }D\left(i,j\right)<d\;\\ 0,\text{otherwise},\; \end{array}\right.$$ (1)

where *j* ∈ KNN(*i*) denotes the *K* instances closest to instance *i*. In this work, we set *K* = 5. *D* (*i*, *j*) indicates the Euclidean distance between two nucleus instances. Thus, we obtain the input for the GCN, the set of nodes and edges *G* = (*V*, *A*). Figure 4 shows the nuclear segmentation results and the graph structure constructed based on these results for three sample patches.

#### 2.3.3 GCN module

The cell structural information used to construct a graph is very suitable for GCN-based feature extraction. To simplify the operation, we use a spatial-based GCN, where the convolution operation is defined as:

$$H^{\left(l+1\right)}=\sigma \left({\tilde{D}}^{-1/2}\tilde{A}{\tilde{D}}^{-1/2}H^{\left(l\right)}W^{\left(l\right)}\right),$$ (2)

in which $H^{\left(l\right)}\in R^{m\times k^{\left(l\right)}}$ denotes the *k* ^(*l*)^-channel features at the *l*th layer, *σ*(.) denotes an activation function, and *W* ^(*l*)^ is the trainable weight matrix of each layer. $\tilde{A}=A+I_{N}$ is the adjacency matrix of the undirected graph with added self-connection, and *I*_*N*_ is the identity matrix.

### 2.4 Semantic feature encoding with CNN

The purpose of semantic feature encoding is to encode the semantic features of an entire image patch, which can be used to mine the overall information contained in the image block and as supplementary information regarding the spatial structure of cells. In this work, we apply VGG16 (Simonyan and Zisserman, 2015) as a feature extractor for the CNN, which consist of a convolutional layer, ReLU, max pooling, and fully connected layer. The model structure is shown as the “CNN module” in Figure 3. The size of the input feature map for each pooling operation is 1/2 that of the previous layer. Finally, the semantic feature encoding the CNN module outputs a feature vector with a length of 1,024.

### 2.5 Multimodal tensor fusion

In many approaches, the features extracted by multiple networks are superimposed onto a set of features through a concatenation operation, followed by convolution operations. However, such approaches are only suitable for extraction of features of the same type. Here, the image semantic features are extracted as a set of feature maps by CNN, and cell structure features are extracted as nodes and edges by GCN. To integrate these multimodal features, we recommend using kronecker product (Zadeh et al., 2017), a feature fusion method for modeling multi-feature interaction. The fusion module combines multimodal tensors through outer product calculation, which can be formulated as:

$$h_{\text{fusion}}=h_{\text{c}}⊗h_{\text{s}},$$ (3)

where ⊗ is the outer product; **h**_c_ and **h**_s_ denote cell graph and semantic features, respectively, and **h**_fusion_ is a differential multimodal tensor formed in a three-dimensional Cartesian space. After aggregating the multimodal tensors, we use a fully connected layer for the next feature operation. In addition, we adopt a gating-based attention mechanism (Arevalo et al., 2017) to limit the fusion proportion of different modal features. For each modality feature **h**_m_, *∀m* ∈ {*c*, *s*}, we learn a linear transformation *W*_*cs*→*m*_ for the weight matrix parameters. The importance score of each feature is defined as $z_{m}=\sigma \left(W_{cs\to m}\cdot \left[h_{c},h_{s}\right]\right)$ . Subsequently, the gated representation **h**_*m*,__gated_ can be calculated as:

$$h_{m,\text{ gated }}=z_{m}*h_{m},\forall m\in \left\{c,s\right\},$$ (4)

where $h_{m}=\mathrm{ReLU}\left(W_{m}\cdot h_{m}\right)$ denotes the feature after activation by ReLU. Through this gated attention mechanism, the expressive ability of each modality feature can be controlled, and the size of the feature space is also reduced before feature fusion.

### 2.6 Loss function

The loss function for LAD-GCN is the standard cross-entropy loss:

$$\underset{P}{\min}L=-\overset{B}{\underset{b=1}{\sum }}p_{b}\log q_{b},$$ (5)

where *A* represents the total number of image patches, and *p*_*b*_ and *q*_*b*_ = *p*_*model*_ (*y*∣*b*) indicate the target labels and the predicted class distribution produced by the model for input *b*, respectively. The whole training process of the network is performed in an end-to-end manner.

## 3 Experiments and results

### 3.1 Implementation details

For the GCN module, we first use a pre-trained nuclei segmentation module to extract various nuclei from each patch, and train the GCN model classifiers. We train the CNN module and GCN module using the Adam optimizer with an initial learning rate of 0.001 and a batch size of 64. For the proposed LAD-GCN, the trained GCN module and CNN module are then fine-tuned for 100 epochs with a learning rate of 0.00001. All our modules were implemented with PyTorch and trained on four NVIDIA Tesla A100 GPUs.

### 3.2 Evaluation metrics

We employed four metrics for performance evaluation of the baseline classification model, GCN module, CNN module, and the proposed LAD-GCN: precision, recall, F1-score, and accuracy. These performance metrics can be understood by considering four terms: true positives (TP), true negatives (TN), false positives (FP), and false negatives (FN). The precision (P) and recall (R) were defined as:

$$P=\frac{TP}{TP+FP},$$ (6)

and

$$R=\frac{TP}{TP+FN}.$$ (7)

We further measured the F1 score (F1S), which combines precision and recall, defined as:

$$F1S=\frac{2TP}{2TP+FP+FN}.$$ (8)

In addition, we calculated the accuracy of five growth patterns, defined as:

$$Accuracy=\frac{TP+TN}{TP+TN+FP+FN}.$$ (9)

### 3.3 Ablation study

To verify the effectiveness of the CNN module and GCN module in lung tumor histopathological image analysis, we conducted an ablation study. The results are shown in Table 1 and Table 2. The CNN module demonstrated greater ability in the analysis of lepidic and acinar growth patterns, whereas the GCN module could better capture the micropapillary structure. Both modules worked well for identifying solid growth patterns, possibly because the tumor cells were densely packed and lacked characteristic patterns of adenocarcinoma. The proposed model (LAD-GCN) fuses semantic features and spatial features. Although it could not achieve optimal results in the analysis of every growth mode, its performance was stable. It achieved an accuracy of 90.35%, which was more than 1.8% better than that of the CNN module, and more than 3.6% better than that of the GCN module.

**TABLE 1:** Effects of each module in our LAD-GCN design. Bold font indicates best result obtained for predictions.

Growth pattern	Methods	CNN	GCN	P (%)	R (%)	F1S (%)
Lepidic	CNN module	*✓*		**90.64**	87.76	89.18
	GCN module		*✓*	84.55	78.55	81.44
	LAD-GCN	*✓*	*✓*	90.26	**89.83**	**90.04**
Acinar	CNN module	*✓*		**89.79**	87.24	88.50
	GCN module		*✓*	83.61	85.98	84.78
	LAD-GCN	*✓*	*✓*	89.27	**90.39**	**89.83**
Papillary	CNN module	*✓*		85.17	79.78	82.39
	GCN module		*✓*	83.33	81.52	82.42
	LAD-GCN	*✓*	*✓*	**87.15**	**85.80**	**86.47**
Micropapillary	CNN module	*✓*		79.56	**89.44**	84.21
	GCN module		*✓*	85.63	89.03	**87.30**
	LAD-GCN	*✓*	*✓*	**86.53**	86.80	86.67
Solid	CNN module	*✓*		98.17	98.02	98.10
	GCN module		*✓*	95.85	97.33	97.07
	LAD-GCN	*✓*	*✓*	**98.34**	**98.78**	**98.56**

**TABLE 2:** Comparison of the performances of each module in terms of accuracy.

Methods	Accuracy (%)
CNN module	88.49
GCN module	86.71
LAD-GCN	**90.35**

**FIGURE 5:**
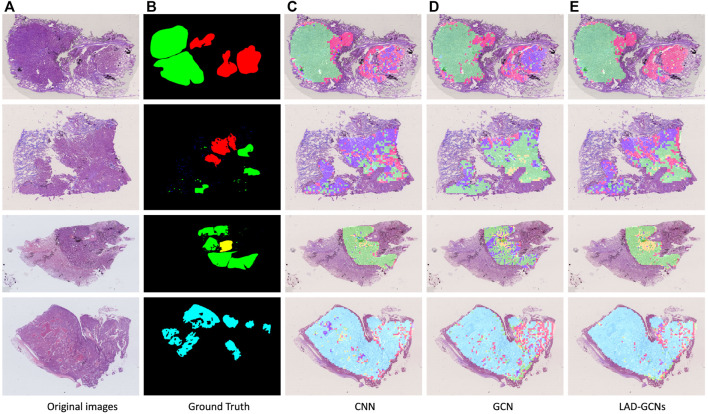
Typical histopathological image analysis results obtained with three networks. Red, green, yellow, blue, and cyan masks represent lepidic, acinar, papillary, micropapillary, and solid growth patterns, respectively. **(A)** Example of histopathological images. **(B)** Ground truth by pathologists. **(C,D,E)** Typical histopathological image analysis results obtained with three networks: **(C)** CNN, **(D)** GCN and **(E)** LAD-GCNs. Red, green, yellow, blue, and cyan masks represent lepidic, acinar, papillary, micropapillary, and solid growth patterns, respectively.

Figure 5 shows the results of histopathological image analysis of four images, which included lepidic, acinar, papillary, micropapillary, and solid growth patterns. The GCN and CNN modules produced very similar masks to the manual ground truth; however, LAD-GCN could still provide a subtle improvement. As shown in the figure, the areas predicted by the deep learning model were often larger than those obtained by manual labeling; this was because manual annotation focused on regions typical of particular growth patterns, whereas the trained deep learning model could predict both typical and atypical growth pattern regions. Pathologists perform semi-quantitative assessments of growth patterns when analyzing histopathological images of lung adenocarcinomas. This process is very dependent on the subjective evaluation of individuals and is difficult to quantify. The trained model could predict the type of growth pattern for each small patch region, enabling quantification of types across the entire histopathological image.

## 4 Discussion and conclusion

In this study, we proposed the LAD-GCN framework, which consists of a GCN module and a CNN module, for the task of lung adenocarcinoma growth pattern prediction. The GCN module captures the spatial structural features between cells, whereas the CNN module captures semantic features of whole patches; these features can be fused to predict growth patterns. In particular, our proposed model showed significantly enhanced performance in lung adenocarcinoma growth pattern prediction tasks. In the future, our goal is to combine image analysis with patient medical records to predict medication and prognostic status, and to apply our LAD-GCN framework to other histopathological WSI analysis tasks such as images of breast, kidney, and brain.

## Data Availability

The original contributions presented in the study are included in the article/supplementary material, further inquiries can be directed to the corresponding author.

## References

[B1] Adnan M. Kalra S. Tizhoosh H. R. (2020). “Representation learning of histopathology images using graph neural networks,” in Proceedings of the IEEE/CVF conference on computer vision and pattern recognition workshops (Seattle, WA: IEEE), 988–989.

[B2] Arevalo J. Solorio T. Montes-y Gómez M. González F. A. (2017). Gated multimodal units for information fusion. Toulon, France: arXiv preprint arXiv:1702.01992.

[B3] Chen R. J. Lu M. Y. Wang J. Williamson D. F. Rodig S. J. Lindeman N. I. (2020). Pathomic fusion: An integrated framework for fusing histopathology and genomic features for cancer diagnosis and prognosis. IEEE Trans. Med. Imaging 41, 757–770. 10.1109/TMI.2020.3021387 PMC10339462 32881682

[B4] Cheng T.-Y. D. Cramb S. M. Baade P. D. Youlden D. R. Nwogu C. Reid M. E. (2016). The international epidemiology of lung cancer: Latest trends, disparities, and tumor characteristics. J. Thorac. Oncol. 11, 1653–1671. 10.1016/j.jtho.2016.05.021 27364315 PMC5512876

[B5] Coudray N. Ocampo P. S. Sakellaropoulos T. Narula N. Snuderl M. Fenyö D. (2018). Classification and mutation prediction from non-small cell lung cancer histopathology images using deep learning. Nat. Med. 24, 1559–1567. 10.1038/s41591-018-0177-5 30224757 PMC9847512

[B6] Fan L. Sowmya A. Meijering E. Song Y. (2021). “Learning visual features by colorization for slide-consistent survival prediction from whole slide images,” in International conference on medical image computing and computer-assisted intervention (Strasbourg, France: Springer), 592–601.

[B7] Ferlay J. Ervik M. Lam F. Colombet M. Mery L. Piñeros M. (2020). Global cancer observatory: Cancer today. Lyon, France: International agency for research on cancer, 1–6.

[B8] Gurcan M. Boucheron L. Can A. Madabhushi A. Rajpoot N. Yener B. (2009). Histopathological image analysis: A review. IEEE Rev. Biomed. Eng. 2, 147–171. 10.1109/RBME.2009.2034865 20671804 PMC2910932

[B9] Henaff O. (2020). “Data-efficient image recognition with contrastive predictive coding,” in International conference on machine learning (Vienna, Austria: PMLR), 4182–4192.

[B10] Hirsch F. R. Scagliotti G. V. Mulshine J. L. Kwon R. Curran W. J. Jr Wu Y.-L. (2017). Lung cancer: Current therapies and new targeted treatments. Lancet 389, 299–311. 10.1016/S0140-6736(16)30958-8 27574741

[B11] Kalra S. Adnan M. Taylor G. Tizhoosh H. R. (2020). “Learning permutation invariant representations using memory networks,” in European conference on computer vision (Glasgow, United Kingdom: Springer), 677–693.

[B12] Kipf T. N. Welling M. (2016). “Semi-supervised classification with graph convolutional networks,” in 5th International Conference on Learning Representations, ICLR 2017, Toulon, France, April 24–26, 2017. Conference Track Proceedings.: arXiv preprint arXiv:1609.02907.

[B13] Li R. Yao J. Zhu X. Li Y. Huang J. (2018). “Graph cnn for survival analysis on whole slide pathological images,” in International conference on medical image computing and computer-assisted intervention (Granada, Spain: Springer), 174–182.

[B14] Mayekar M. K. Bivona T. G. (2017). Current landscape of targeted therapy in lung cancer. Clin. Pharmacol. Ther. 102, 757–764. 10.1002/cpt.810 28786099

[B15] Muja M. Lowe D. G. (2009). Fast approximate nearest neighbors with automatic algorithm configuration. VISAPP 2 (1), 2.

[B16] Noorbakhsh J. Farahmand S. Namburi S. Caruana D. Rimm D. Soltanieh-ha M. (2020). Deep learning-based cross-classifications reveal conserved spatial behaviors within tumor histological images. Nat. Commun. 11, 6367. 10.1038/s41467-020-20030-5 33311458 PMC7733499

[B17] Ronneberger O. Fischer P. Brox T. (2015). “U-net: Convolutional networks for biomedical image segmentation,” in International Conference on Medical image computing and computer-assisted intervention (Munich, Germany: Springer), 234–241.

[B18] Šarić M. Russo M. Stella M. Sikora M. (2019). “Cnn-based method for lung cancer detection in whole slide histopathology images,” in 2019 4th international conference on smart and sustainable technologies (SpliTech) (Bol and Split, Croatia: IEEE), 1–4.

[B19] Simonyan K. Zisserman A. (2015). “Very deep convolutional networks for large-scale image recognition,” in 3rd International Conference on Learning Representations, ICLR 2015, San Diego, CA, May 7–9, 2015. Conference Track Proceedings.: arXiv preprint arXiv:1409.1556.

[B20] Szegedy C. Vanhoucke V. Ioffe S. Shlens J. Wojna Z. (2016). “Rethinking the inception architecture for computer vision,” in 2016 IEEE/CVF conference on computer vision and pattern recognition (CVPR) (Las Vegas, NV: IEEE), 2818–2826.

[B21] Travis W. D. Brambilla E. Nicholson A. G. Yatabe Y. Austin J. H. Beasley M. B. (2015). The 2015 world health organization classification of lung tumors: Impact of genetic, clinical and radiologic advances since the 2004 classification. J. Thorac. Oncol. 10, 1243–1260. 10.1097/JTO.0000000000000630 26291008

[B22] Travis W. D. Brambilla E. Noguchi M. Nicholson A. G. Geisinger K. R. Yatabe Y. (2011). International association for the study of lung cancer/american thoracic society/european respiratory society international multidisciplinary classification of lung adenocarcinoma. J. Thorac. Oncol. 6, 244–285. 10.1097/JTO.0b013e318206a221 21252716 PMC4513953

[B23] Van Griethuysen J. J. Fedorov A. Parmar C. Hosny A. Aucoin N. Narayan V. (2017). Computational radiomics system to decode the radiographic phenotype. Cancer Res. 77, e104–e107. 10.1158/0008-5472.CAN-17-0339 29092951 PMC5672828

[B24] Wang S. Rong R. Yang D. M. Fujimoto J. Yan S. Cai L. (2020). Computational staining of pathology images to study the tumor microenvironment in lung cancer. Cancer Res. 80, 2056–2066. 10.1158/0008-5472.CAN-19-1629 31915129 PMC7919065

[B25] Xiao W. Jiang Y. Yao Z. Zhou X. Lian J. Zheng Y. (2021). Polar representation-based cell nucleus segmentation in non-small cell lung cancer histopathological images. Biomed. Signal Process. Control 70, 103028. 10.1016/j.bspc.2021.103028

[B26] Xie E. Sun P. Song X. Wang W. Liu X. Liang D. (2020). “Polarmask: Single shot instance segmentation with polar representation,” in Proceedings of the IEEE/CVF conference on computer vision and pattern recognition (Seattle, WA: IEEE), 12193–12202.

[B27] Xie S. Tu Z. (2015). “Holistically-nested edge detection,” in Proceedings of the IEEE international conference on computer vision (Santiago, Chile: IEEE), 1395–1403.

[B28] Yu K.-H. Wang F. Berry G. J. Ré C. Altman R. B. Snyder M. (2020). Classifying non-small cell lung cancer types and transcriptomic subtypes using convolutional neural networks. J. Am. Med. Inf. Assoc. 27, 757–769. 10.1093/jamia/ocz230 PMC7309263 32364237

[B29] Zadeh A. Chen M. Poria S. Cambria E. Morency L.-P. (2017). “Tensor fusion network for multimodal sentiment analysis,” in 2017 conference on empirical methods in natural language processing (Copenhagen, Denmark: Association for Computational Linguistics).

[B30] Zhou Y. Graham S. Alemi Koohbanani N. Shaban M. Heng P.-A. Rajpoot N. (2019). “Cgc-net: Cell graph convolutional network for grading of colorectal cancer histology images,” in Proceedings of the IEEE/CVF international conference on computer vision workshops (Seoul, Korea: IEEE).

